# Endoscopic ultrasound-guided choledochoduodenostomy using a novel, ultra-stiff, high-sliding guidewire and a dumbbell-shaped metal stent

**DOI:** 10.1055/a-2241-2030

**Published:** 2024-02-15

**Authors:** Tadahisa Inoue, Mayu Ibusuki, Rena Kitano, Kiyoaki Ito

**Affiliations:** 1 12703 Department of Gastroenterology, Aichi Medical University, Nagakute, Japan

Endoscopic ultrasound-guided choledochoduodenostomy (EUS-CDS) has potential as a first-line drainage method for malignant distal biliary obstruction. Several randomized controlled trials revealed that EUS-CDS with lumen-apposing metal stent (LAMS) have higher technical success rates and shorter procedure times than conventional transpapillary drainage 1 2 . However, thin 6-mm diameter LAMSs are frequently used due to their larger flanges, and these stents are associated with adverse events such as biliary wall compression and duodenobiliary reflux, as well as being expensive 3 . Conversely, EUS-CDS using conventional metal stents is associated with a risk of bile leakage due to the need for fistula dilation, as well as stent migration. Herein, we report a new EUS-CDS technique using a novel, ultra-stiff, high-sliding guidewire and a dumbbell-shaped metal stent.

The dumbbell-shaped metal stent (BONASTENT M-Intraductal; Standard Sci-Tech Inc., Seoul, Korea) has a diameter of 8 mm at the central portion and 12 mm at the proximal and distal portions 4 , providing antimigration properties. The 0.035-inch novel guidewire (SeekMaster Hard; Piolax Medical Devices, Kanagawa, Japan) consists of a thick (0.7 mm in diameter), high-rigidity, nickel-titanium core wire. The polytetrafluoroethylene-coated surface of the wire with “ridge-processing” reduces the contact area and friction with the metal stent delivery system, while increasing the sliding properties and achieving high device followability and insertability, thereby eliminating the need for fistula dilation ( Fig. 1 ).

**Fig. 1:**
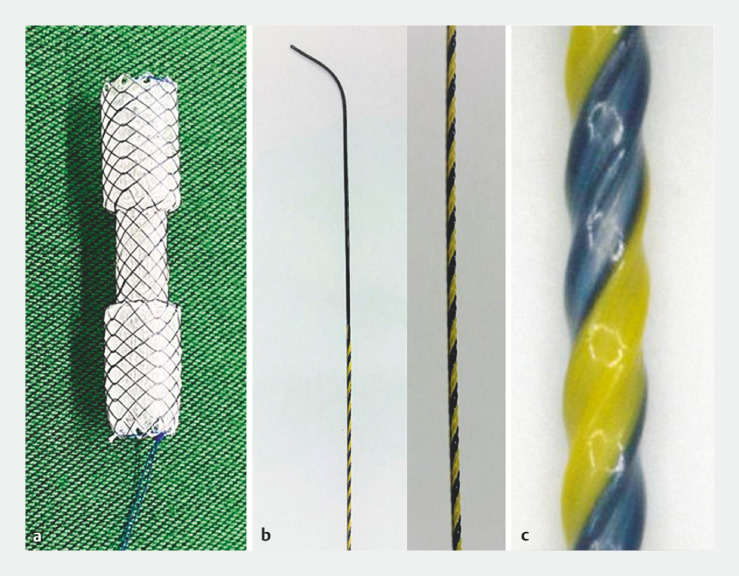
The stent and guidewire. **a** The dumbbell-shaped metal stent (BONASTENT M-Intraductal; Standard Sci-Tech Inc., Seoul, Korea) consists of a diameter of 8 mm in the central portion (15 mm length) and 12 mm in the proximal and distal portions (17.5 mm length, each), providing antimigration properties. **b** The 0.035-inch novel guidewire (SeekMaster Hard; Piolax Medical Devices, Kanagawa, Japan) consists of a thick (0.7 mm in diameter), high-rigidity, nickel-titanium core wire. **c** The surface of the wire is coated with polytetrafluoroethylene with “ridge-processing,” which reduces the contact area and friction with the metal stent delivery system, increasing sliding properties, and achieving extremely high device followability and insertability, thereby eliminating the need for fistula dilation.

A 73-year-old man with pancreatic cancer developed obstructive jaundice due to malignant distal biliary obstruction. A linear-array echoendoscope was inserted, and the common bile duct was punctured from the duodenum using a 19-G needle. The novel guidewire was inserted through the needle and advanced to the intrahepatic bile duct. The dumbbell-shaped metal stent delivery system was inserted over the guidewire without fistula dilation; the metal stent was deployed from the common bile duct to the duodenum ( Fig. 2 , Video 1 ). The procedure time was 5 minutes, with no adverse events. The stent was patent until the patient’s death, with no stent dysfunction including migration.

**Fig. 2:**
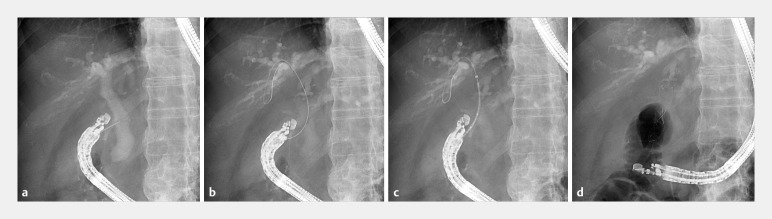
Fluoroscopic views. **a** A linear-array echoendoscope was inserted, and the common bile duct was punctured from the duodenum using a 19-G needle. **b** The novel guidewire was inserted through the needle and advanced to the intrahepatic bile duct. **c** Subsequently, the dumbbell-shaped metal stent delivery system was inserted over the guidewire without fistula dilation. **d** The metal stent was deployed from the common bile duct to the duodenum.

Endoscopic ultrasound-guided choledochoduodenostomy using the novel, ultra-stiff, high-sliding guidewire and the dumbbell-shaped metal stent.Video 1

This technique overcomes the shortcomings of EUS-CDS with LAMS, while allowing the same ease of application.

Endoscopy_UCTN_Code_TTT_1AS_2AD

## Notes

Endoscopy E-Videos https://eref.thieme.de/e-videos
*E-Videos* is an open access online section of the journal *Endoscopy* , reporting on interesting cases and new techniques in gastroenterological endoscopy. All papers include a high-quality video and are published with a Creative Commons CC-BY license. Endoscopy E-Videos qualify for HINARI discounts and waivers and eligibility is automatically checked during the submission process. We grant 100% waivers to articles whose corresponding authors are based in Group A countries and 50% waivers to those who are based in Group B countries as classified by Research4Life (see: https://www.research4life.org/access/eligibility/ ). This section has its own submission website at https://mc.manuscriptcentral.com/e-videos .
